# Testing the Global Malaise Trap Program – How well does the current barcode reference library identify flying insects in Germany?

**DOI:** 10.3897/BDJ.4.e10671

**Published:** 2016-12-01

**Authors:** Matthias F. Geiger, Jerome Moriniere, Axel Hausmann, Gerhard Haszprunar, Wolfgang Wägele, Paul D.N. Hebert, Björn Rulik

**Affiliations:** ‡ Zoologisches Forschungsmuseum Alexander Koenig, Bonn, Germany; § SNSB-Zoologische Staatssammlung, München, Germany; | Centre for Biodiversity Genomics, Biodiversity Institute of Ontario, University of Guelph, Guelph, Canada

**Keywords:** DNA barcoding, arthropods, reverse taxonomy, BIN discordance, biomonitoring

## Abstract

**Background:**

Biodiversity patterns are inherently complex and difficult to comprehensively assess. Yet, deciphering shifts in species composition through time and space are crucial for efficient and successful management of ecosystem services, as well as for predicting change. To better understand species diversity patterns, Germany participated in the Global Malaise Trap Program, a world-wide collection program for arthropods using this sampling method followed by their DNA barcode analysis. Traps were deployed at two localities: “Nationalpark Bayerischer Wald” in Bavaria, the largest terrestrial Natura 2000 area in Germany, and the nature conservation area Landskrone, an EU habitats directive site in the Rhine Valley. Arthropods were collected from May to September to track shifts in the taxonomic composition and temporal succession at these locations.

**New information:**

In total, 37,274 specimens were sorted and DNA barcoded, resulting in 5,301 different genetic clusters (BINs, Barcode Index Numbers, proxy for species) with just 7.6% of their BINs shared. Accumulation curves for the BIN count versus the number of specimens analyzed suggest that about 63% of the potential diversity at these sites was recovered with this single season of sampling. Diversity at both sites rose from May (496 & 565 BINs) to July (1,236 & 1,522 BINs) before decreasing in September (572 & 504 BINs). Unambiguous species names were assigned to 35% of the BINs (1,868) which represented 12,640 specimens. Another 7% of the BINs (386) with 1,988 specimens were assigned to genus, while 26% (1,390) with 12,092 specimens were only placed to a family. These results illustrate how a comprehensive DNA barcode reference library can identify unknown specimens, but also reveal how this potential is constrained by gaps in the quantity and quality of records in BOLD, especially for Hymenoptera and Diptera. As voucher specimens are available for morphological study, we invite taxonomic experts to assist in the identification of unnamed BINs.

## Introduction

Initiated in 2012 by the Centre for Biodiversity Genomics at the Biodiversity Institute of Ontario (BIO), the Global Malaise Trap Program (GMTP) is a collaboration involving more than 30 international partners. It aims to provide an overview of arthropod diversity by coupling the large-scale deployment of Malaise traps with the use of specimen-based DNA barcoding to assess species diversity. Because arthropods comprise the overwhelming majority of species in terrestrial habitats (Kremen et al. 1993) and possess tremendous trait variation, it would be a quantum leap for ecology and the modelling of biodiversity change if their responses to environmental change could be assessed (Timms et al. 2012). Until recently, this was impossible because no systematic approach was available to rapidly identify and quantify arthropod diversity, a barrier which prevented the detection of shifts in species composition in response to habitat disturbance (Samways 1993). Prior studies have employed large-scale Malaise Trap (Malaise 1937) deployments to advance the monitoring and assessment of arthropod diversity. For example, the Swedish Malaise Trap project placed 61 traps at 44 localities from 2003 to 2006 (Karlsson et al. 2005, Ronquist 2010). Analysis of these collections has so far increased the Swedish insect fauna by more than 1,900 species, including several hundred new to science (Ronquist 2010). However, the sorting and identification of the estimated 40 million specimens through morphological approaches will occupy biologists for many decades. The emergence of DNA barcoding as a method to rapidly sort and objectively differentiate species (Hebert et al. 2003) and its global adoption (Hebert et al. 2009, Miller et al. 2016, Taylor and Harris 2012) has provided a new tool for assessing arthropod diversity for diverse applications including the management of natural resources. The GMTP combines the power of DNA barcoding to discriminate species with the capacity of Malaise traps to capture a broad range of arthropods to support the large-scale sampling programs needed for time- and cost-efficient assessment of regional biodiversity.

The arthropod fauna of Germany is thought to include about 38,000 species, with about 1/3 being abundant or very common, while another third are very local and/or rare, and the final third are very rare, being collected only a few times each century and usually with Red List status if recognized at all. This overall abundance pattern implies that a DNA barcode reference library with 50-60% species coverage should allow for the re-identification of some 80-95% of the specimens encountered in real samples, a sufficient level of resolution for most applications. Although Malaise traps are particularly effective in capturing flying insects (especially Diptera and Hymenoptera), they also collect other arthropods, including wingless species, that crawl into them. Because of their high taxonomic diversity, Malaise trap catches are ideal for testing the adequacy of current taxonomic coverage in the DNA barcode reference library. To evaluate the efficacy of this approach, we employed a reverse taxonomy approach (Markmann and Tautz 2005) using the BIN (Barcode Index Number) system as a basis for identifying specimens collected by the two Malaise traps deployed in Germany. The BIN system is a DNA-based registry for animal diversity, which was established on the Barcode of Life Datasystem (BOLD) as a tool to delineate species proxies (Ratnasingham and Hebert 2013). Based on a combination of genetic distance (2.2% *ab initio*) and subsequent refinement using Markov clustering, it has proven extremely valuable in identifying specimens collected in large-scale biodiversity inventories (Aagaard et al. 2016, Telfer et al. 2015, Wirta et al. 2015) and as an additional character for comparing traditional taxonomy concepts in, e.g. bees (Schmidt et al. 2015), beetles (Oba et al. 2015, Hendrich et al. 2014), bugs (Raupach et al. 2014), butterflies and moths (Hausmann et al. 2013, Huemer et al. 2014), fishes (Knebelsberger et al. 2014a, Knebelsberger et al. 2014b), Hemiptera (Gwiazdowski et al. 2015), Neuroptera (Morinière et al. 2014), and reptiles and amphibians (Hawlitschek et al. 2015). Comparisons of the performance of the BIN system with other approaches for species delimitation (Kekkonen and Hebert 2014, Collins and Cruickshank 2014) indicated that it is superior in term of computational performance, but also that it might not work equally well in all animal groups, as for example illustrated in *Tanytarsus* nonbiting midges (Lin et al. 2015). However, its primary advantage is the fact that all BINs are registered on BOLD, enabling these records to assign unidentified specimens to a taxonomic entity. As a byproduct, the reverse taxonomy approach highlights gaps in the reference library as BOLD fails to deliver an identification in these cases, providing motivation for focused taxonomic studies to fill these gaps. Ideally these new records are connected to voucher specimens that are then identified as exemplified by a fly species encountered in the present analyses (Reimann and Rulik 2015). Moreover, taxonomic assignments are facilitated, because specimens that are not assigned to a species are often assigned to the correct genus, and routinely to the correct subfamily or family. With this information on taxonomic placement and with the voucher specimen, it is far simpler to make a species assignment than beginning the effort at identification without any advance knowledge.

We used samples from the two Malaise traps to assess the current taxonomic coverage of the DNA barcode reference library by assessing the percentage of BINs that were automatically assigned with an unambiguous species-, genus-, or family level taxonomy. In our definition a BIN’s identification is classified unambiguous if it contains specimens with only one taxon name of the same rank (species, genus, etc.). For this purpose we used the BOLD “discordance report” without further inspecting the discordant BINs, which is a conservative approach. Although it overestimates gaps in the reference libraries due to “noise” (obvious errors and misidentifications leading to mismatches at higher systematic levels), we opted for this strategy, because it mimics the situation in metabarcoding studies: large numbers of sequence clusters are generated and automatically given a taxonomic placement that cannot be manually checked on an individual basis. This is often due to a lack of taxonomic expertise that would be necessary to decide which taxon is the correct one in cases where more than one is retrieved as a “hit” from the BOLD identification query. We also tried to ascertain if taxonomic coverage in the reference database is biased towards organisms which occur frequently and in high abundance. We further compared the α-diversity between the two locations from May to September, using BIN assignments as a species proxy to describe shifts in diversity over the course of the year.

## Materials and Methods

**Malaise Trap details**

As part of the GMTP, two Malaise traps were deployed in Germany, one in southeast Germany in the Nationalpark Bayerischer Wald in 2012 (GMTPE: 11.3 km N of Grafenau, conifer-dominated mountain forest, 842m asl, N 48.95090 E 13.42199), which is the largest terrestrial protected natural reserve in Germany, and, combined with the neighbouring Czech Šumava National Park, part of the largest transboundary protected area in central Europe. The sample location can be described as natural forest (*Luzulo nemorosae* -*Abietetum*) in a frost-pocket with stand replacing disturbance by spruce bark beetles, close to the 'Racheldiensthütte'. The second trap was installed in 2013 in the western part of Germany, situated in the north adjacent to the Upper Middle River Rhine Valley (GMTPZ: 5.0 km E of Bad Neuenahr-Ahrweiler, 'Landskrone', Oberwinterer terrace- and hill country, 194m asl, N 50.55600 E 7.17000). The specific trapping site can be described as follows: southerly exposure, on the edge of a sandy bluff surrounded by xeric grassland in proximity to a deciduous coppice (‘Niederwald’). The vegetation is eradicated annually due to St. Martin’s fires, which occur every November, followed by pioneer vegetation again. The two sample locations are separated by about 485 kilometers.

The collection bottles for each Malaise trap were emptied weekly or biweekly and filled with 500 ml of fresh 80% (GMTPE) or 96% (GMTPZ) laboratory EtOH. Subsequent analyses only consider the arthropod diversity observed in traps between May and September as this was the interval during which sampling was continuous at both sites.

**Specimen Sorting and DNA Barcoding**

Individuals from each trap were sorted and DNA barcoded following standard protocols for the Global Malaise Trap Program (http://globalmalaise.org/about) without taxonomic assignment below an ordinal level. Large-bodied specimens were pinned and stored as vouchers after a leg was removed for DNA extraction while small-bodied specimens were placed directly in 96-well microplates and were recovered after DNA extraction and then stored individually in 96% EtOH. Standard protocols (http://ccdb.ca/resources.php) were used to recover the DNA barcode region, with the exception that the PCR products were only sequenced unidirectionally. All meta- and sequence data were uploaded to the online Barcode of Life Database (www.boldsystems.org) into the public project containers GMTPE (projects: GMGRA, GMGRD, GMGRE, GMGRC, GMGRB, GMGRF, GMGRG, GMGRH, GMGRI) and GMTPZ (projects: GMGMH, GMGMJ, GMGMA, GMGMK, GMGMI, GMGMG, GMGMF, GMGMM, GMGML, GMGMB, GMGME, GMGMN, GMGMC, GMGMD). Based on the comparison of the DNA barcode records to data already on BOLD, each sequence was assigned a new or known BIN. As well, one or more representatives of each species or BIN were imaged (available in each project container). Finally, representatives of all new BINs were bidirectional sequenced to ensure that these records were in full compliance with the barcode standard (Hanner 2009). The voucher specimens were then returned to Germany where they are permanently stored in the scientific collections at SNSB-ZSM (Bavarian State Collection of Zoology, GMTPE) and ZFMK (Zoological Research Museum Alexander Koenig, GMTPZ). All vouchers are available from the authors upon request. All DNA sequences and specimen metadata can be viewed and downloaded from the dataset DS-765MFBAY (http://dx.doi.org/10.5883/DS-765MFBAY) and DS-633MF8 (http://dx.doi.org/10.5883/DS-633MF8).

**Data Analysis**

All specimens with a full or partial DNA barcode were assigned to a new or an existing BIN, so automated identifications based on BIN membership were annotated by the BOLD-ID engine. BINs new to the barcode library are assigned a taxonomic level above the species (genus or family, usually). This is also the case if multiple species names occur in the same BIN, even if these cases of BIN-sharing do not involve specimens from the same country or continent. As BOLD is constantly growing and BIN assignments are dynamic, all data from the two projects were downloaded on August 27^th^ 2015 and all analyses reflect the state of knowledge at this time. This is important because the addition of new sequences to the database can alter BIN assignments, either removing some members of a BIN, or merging formerly separate BINs into one (e.g., Hausmann et al. 2013). Furthermore, the database is constantly updated by experts who revise the taxonomy assignment for a BIN or provide a species name for unnamed BINs. Thus, as the reference database for German arthropods increase, the estimates for the proportions of shared taxa between the Malaise traps and between consecutive months will show small changes. In order to assess the taxonomic coverage of the DNA barcode reference library we generated a BIN discordance report on BOLD. This report listed all specimens with their identification based on named BINs, i.e. BINs which contain one or more individuals with an authoritative identification to a Linnean binomen. Because of the large amount of data, we used a spreadsheet-based strategy and only chose one representative per BIN (the one with the longest COI sequence) and generated a list of sample IDs which can easily be copied into the search field on BOLD. By avoiding redundant BIN entries, this approach saves considerable computational time.

We evaluated the sampling effort and proportion of diversity in each trap with accumulation curves for number of BINs versus the number of DNA barcodes separately for the GMTPE and GMTPZ samples. These analyses employed EstimateS 9.1.0 with 100 randomizations and a total extrapolation by a factor of 3 (Colwell 2013). Venn diagrams provided an excellent way to visualize the intersection of BINs between consecutive months and the two sample locations. We used an open access tool to upload the set of BINs for each month and for each order from the two locations. Duplicates in a category (month or trap) were then removed before the diagram was drawn and graphically manipulated in Adobe Illustrator®.

## Data resources

All DNA sequences and specimen metadata can be viewed and downloaded from the dataset found at http://dx.doi.org/10.5883/DS-GMTGER.

All records that represented BINs which were new to BOLD are compiled in new datasets, and can be retrieved from BOLD under the code DS-765MFBAY (http://dx.doi.org/10.5883/DS-765MFBAY) and DS-633MF8 (http://dx.doi.org/10.5883/DS-633MF8).

Further sources of primary data can be found in the supplemetary material.

## Results

A total of 29,490 (GMTPE) and 16,002 (GMTPZ) specimens collected from May through September were selected for barcode analysis. From these specimens, 23,752 (80.5%, GMTPE) and 13,524 (84.5%, GMTPZ) delivered a full or partial DNA barcode (>285 bps; mean=595 +/- 41 bps SD), enough data to permit their assignment to a BIN. In total, 2,540 BINs were represented in the sample from the Nationalpark Bayerischer Wald (GMTPE) while the Landskrone sample (GMTPZ) included 2,761 BINs. The GMTPE samples contained 3,749 individuals that represented 765 BINs that were new to BOLD (August 2015). Similarly, the GMTPZ samples included 1,980 specimens that represented 633 BINs, which were new to BOLD. These records are compiled in new datasets, which can be retrieved from BOLD under the code DS-765MFBAY (http://dx.doi.org/10.5883/DS-765MFBAY) and DS-633MF8 (http://dx.doi.org/10.5883/DS-633MF8).

**Figure 1.**
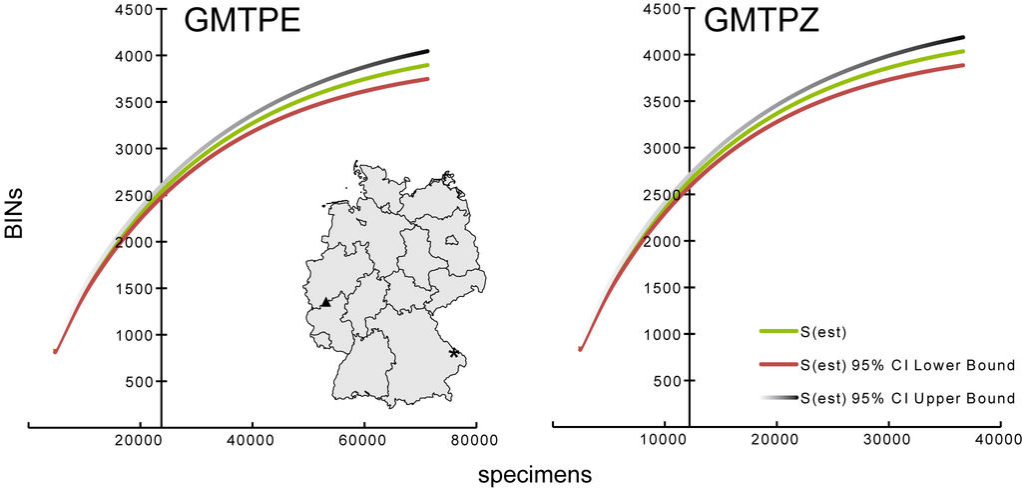
Accumulation curves showing the number of BINs versus the number of specimens analyzed from the two sample locations based on collections from May to September. The map illustrates the location of the two Malaise traps within Germany. Note that the y-axis cuts at the actual number of specimens analysed. Asterik = GMTPE: Nationalpark Bayerischer Wald Triangle = GMTPZ: Middle River Rhine Valley

The accumulation curves for BINs versus the number of analysed specimens (Fig. 1) suggests that slightly less than two thirds (approx. 62.5%) of the expected arthropod diversity has been captured. Additionally, both traps possessed a high proportion of BINs that were either represented by singletons or that were only known from that location (GMTPE: 45% & 54% and GMTPZ: 48% & 56%). Despite different sampling effort (analysed specimens), a similar number of genetic clusters (BINs) has been detected, although the GMTPZ sample contained 8.7% more BINs. Just 407 BINs were shared by the two locations, meaning the overlap was just 7.6%.

**Figure 2.**
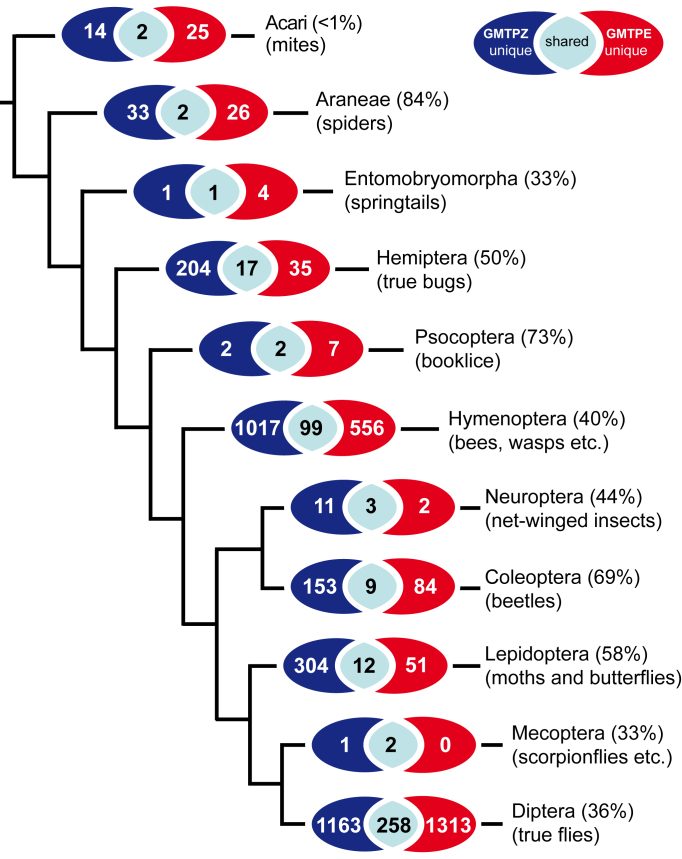
BIN-overlap between the two sample locations on order level; percentage of BINs identified to genus or species level in parentheses. Tree representing a current estimate of arthropod relationships (from Misof et al. 2014), orders without overlap between the two traps have been omitted.

With regard to the distribution of all BINs categorized at order-level, there were similar and low numbers of species belonging to non-target groups (e.g., mites, spiders, booklice) versus much higher species numbers in groups targeted with a Malaise trap (e.g., flies, butterflies and moths, bees and wasps; Fig. 2). The percentage of shared BINs between the two locations in these species-rich groups ranged from a low of 3.3% in Lepidoptera to a high of 9.4% in Diptera (Fig. 2). Among all insect orders, species diversity was, by far, the highest in Diptera which were represented by 2,734 BINs, with slightly more than half (51.6%) of all BINs and 70.3% of all individuals (26,189) that were analyzed.

**Figure 3.**
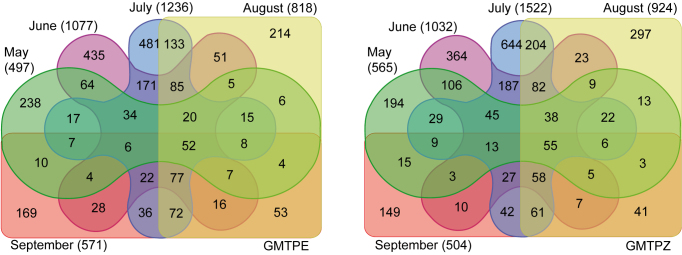
Total number of BINs per month (in parentheses), private BINs per month (in non-overlaid colored fields), and BIN overlap during the course of the summer and between months. For example, the highest number of private BINs not present in any other month occurred in July (481 & 644), while the highest number of shared BINs occurred between June and July (171 & 187) and the number of BINs present throughout the sampling period was 52 and 55; GMTPE left and GMTPZ right.

Analysis of temporal variation in BIN representation revealed a similar overall pattern for both locations with an increasing number of species from May to June and July followed by a decrease in the last two months (Fig. 3).

**Figure 4.**
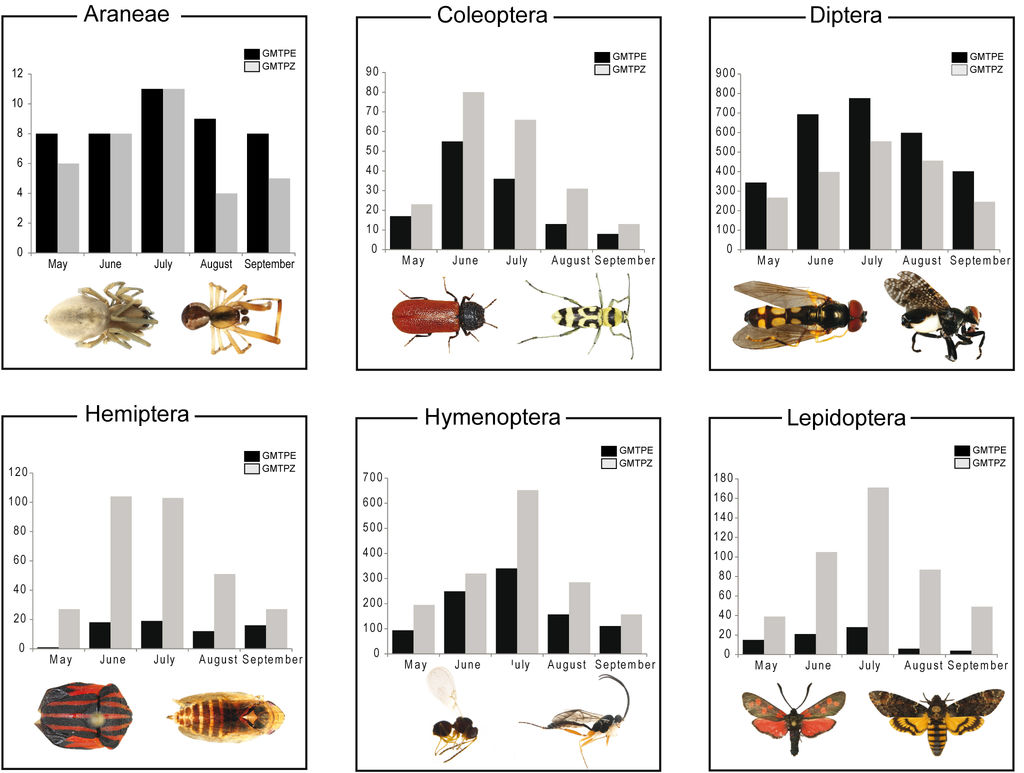
Number of BINs for six major arthropod orders versus month for the two sample locations, GMTPE in the Nationalpark Bayerischer Wald and GMTPZ in the Middle River Rhine Valley.

The same temporal pattern was generally evident for six major orders (Araneae, Coleoptera, Diptera, Hemiptera, Hymenoptera, Lepidoptera), with a steep decline of species numbers between July and August (Fig. 4). Hemiptera from the Bavarian National Park trap (GMTPE) deviated from this general pattern, while Coleoptera diversity peaked in June instead of July (Fig. 4). The proportion of shared BINs in consecutive months was similar for both traps, ranging from 3.8% (GMTPE) and 2.9 % (GMTPZ) shared in August and September to 7.4% in June and July (GMTPE) and 8.3% in July and August (GMTPZ) (Fig. 3). The greatest proportion of unique BINs was observed in the May sample from GMTPE with 238 BINs (47.9%) not observed in any other month. In the other trap there was a peak of unique BINs in July, with 644 BINs (42.3%) not detected in any other month in GMTPZ samples.

Table 1 and Table 2 list the 10 most common BINs from each trap with persistence throughout the whole season, led by an unidentified species of phorid fly in the GMTPE sample (as of October 2015) and another fly (*Coenosia testacea*) in GMTPZ. In order to demonstrate the dynamics of the BIN algorithm and BOLD we include the most recent taxonomic identifications of the 10 most common BINs one year later (Tables 1, 2).

**Figure 5.**
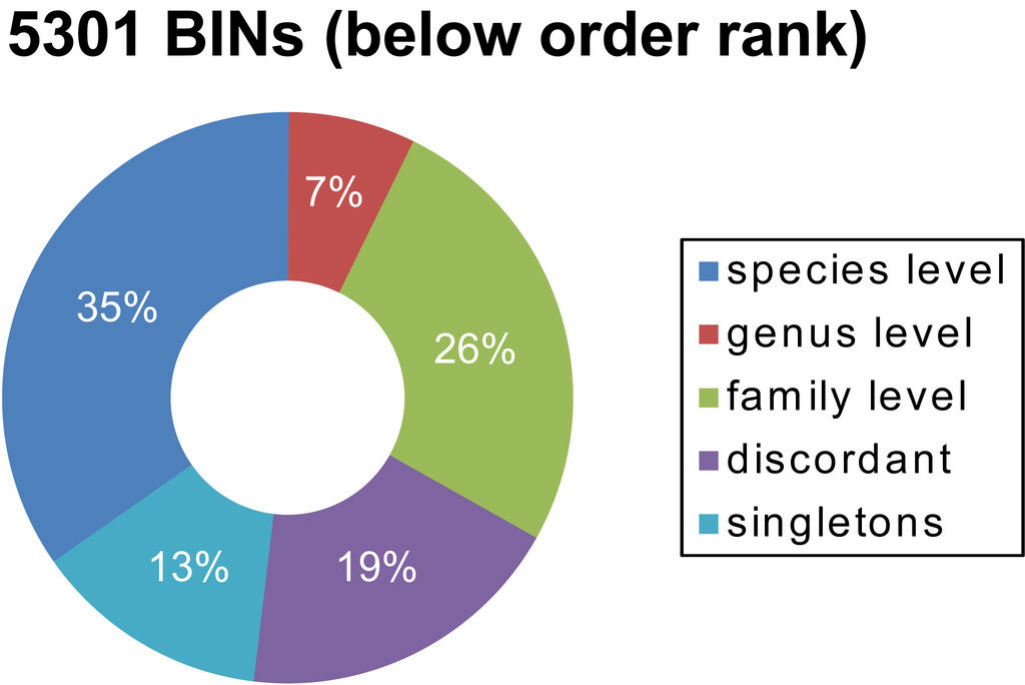
Summary of the BOLD BIN-discordance report for all BINs from both traps assigned to at least an order level. Depicted is the percentage of all BINs, which were grouped into taxonomic homogeneous or heterogeneous BINs and the percentage of BINs represented by only one specimen.

The BIN discordance report (Suppl. material 1) for the combined data from both traps revealed that 35% (1,868 /5,301) of the BINs could be unambiguously identified to a species and that these BINs included 12,640 individuals. Another 7% were identified to a genus (386 BINs, 1,988 individuals), while 26% were placed to a family level (1,390 BINs, 12,092 individuals) (Fig. 5). Among all BINs, 13% (718) were represented by single individuals new to BOLD, while the final 19% of the BINs (9,838 specimens) belonged to BINs that possessed discordant taxonomic assignments. The 939 discordant BINs were further divided into i) 19 with conflicts at an order- or class-level, ii) 44 with a family conflict, iii) 230 with a genus conflict, iv) 209 with a species conflict, and v) 434 with inadequate taxonomy at a genus- or species-level (‘No taxonomy’).

As depicted in Fig. 2, from 36% (Diptera) to 70% (Coleoptera) of the BINs belonging to species-rich orders were unambiguously assigned to a genus or species using the semi-automated BOLD annotation feature. By comparison 40% of Hymenoptera and 58% of Lepidoptera were automatically annotated with a genus or species identification.

## Discussion

**Figure 6.**
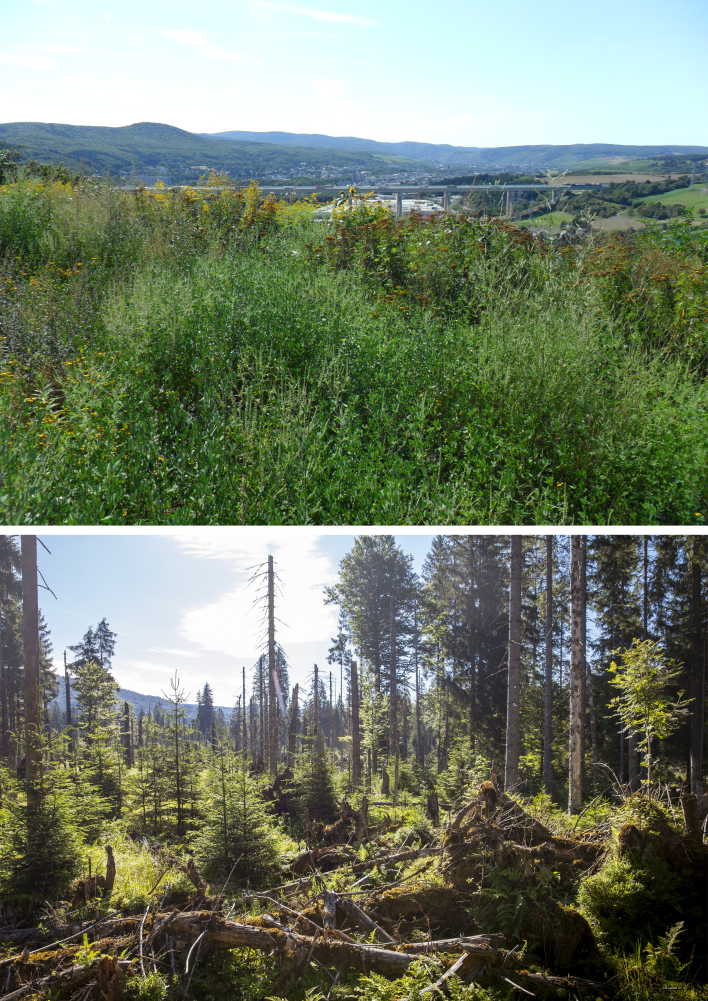
Picture of the direct vicinity of the two Malaise trap locations. Top: GMTPZ adjacent to the Middle River Rhine Valley, view towards the River Ahr valley (by GBOL Team); bottom: GMTPE in the Nationalpark Bayerischer Wald (by Bernhard Huber).

This study did not aim to generate conclusions concerning ecological differences between the two sample locations because they represent fundamentally different types of habitat (cf. Fig. 6) and the samples were collected in different years. Moreover, because of its earlier sampling, BINs for the GMTPE were available for an additional year, providing more time for their re-evaluation and curation. Given these constraints, we do not try to explain the 2-6 fold higher BIN counts for Lepidoptera, Coleoptera, Hymenoptera and Hemiptera at GMTPZ. Instead, we focus on illustrating the potential of the present workflow to advance understanding of terrestrial insect diversity, particularly if conducted in a comparable context (temporal and/or habitat-wise).

The reverse taxonomy approach enabled by the BIN system or by use of the BLAST function against NCBI GenBank records is the standard for large-scale biomonitoring, which employ NGS-based methods to examine environmental samples (e.g., Cristescu 2014, Yu et al. 2012). However, we emphasize that the results generated from such analyses depend crucially on the reference sequence database used to generate identifications. As a result, it is important to track its improvement through time. Metabarcoding studies, based on the analysis of DNA extracts from homogenized mass samples (‘biodiversity soup’), do not enhance the reference database and cannot be validated, because it is usually not possible to re-extract DNA from the bulk sample or single specimens. While metabarcoding is improving our capacity to address ecological questions in work targeting specific communities (e.g., Arrizabalaga-Escudero et al. 2015, Mollot et al. 2014), taxonomic assignments for most sequences is only possible in environments with low taxonomic diversity. For example, because species diversity is relatively low in freshwater ecosystems, metabarcoding has been quite successful in these settings (Elbrecht and Leese 2015, Dowle et al. 2015, Valentini et al. 2016). However, similar success on terrestrial environments is currently limited to settings such as the high arctic (Wirta et al. 2015). Because the present study reveals that the barcode library is only currently sufficient to generate species level identifications for 35% of the specimens collected in Germany, we urge its efforts to extend the reference library. Viewed from this regard, specimens gathered by the GMTP represent a major resource for library expansion, if their taxonomic annotation is improved.

This study indicates that taxonomic coverage achieved during the national initiatives BFB and GBOL has led to considerable progress, but that it has been insufficient to create the reference library needed to identify the *majority* of small flying insects to genus or species level. Additional efforts are required to resolve taxonomic inconsistencies. If we define the biggest gaps in the reference library as those groups with the smallest proportion of unambiguous suprafamily-level taxonomy assigned to their BINs in Germany, then – not surprisingly – Diptera and Hymenoptera stand out. The two groups also contributed most individuals to the samples, and their members are notoriously difficult to identify. On the positive side, the *ad hoc* identification success was good (50-84%) for beetles, butterflies, moths, true bugs and spiders, and there are strong prospects that it will improve in the future due to the large community of taxonomists working on these groups. In fact, the identification success is already higher when some additional effort is invested in the examination of the discordant BINs (e.g. nearly 100% for Lepidoptera and Coleoptera collected in the GMTPE).

Most biologists and even members of the public can recognize and correct gross errors in BOLD, such as those where a beetle shares a BIN with a spider, cases that reflect analytical or data entry errors. Cases where a BIN includes specimens assigned to several families can often be resolved by researchers working on the order by examining photos of the underlying voucher specimens on BOLD. However, cases where a BIN contains more than one valid genus or species name represented almost half of the discordant BINs in the present study and these are much more difficult to resolve. Detailed taxonomic expertise is often essential to distinguish between cases of synonymy or misidentification, with constraints introduced by hybridization or incomplete lineage sorting. Demonstrating the latter complexities should ideally incorporate the analysis of nuclear genetic markers. On the other hand, resolving the discordant BINs tagged with ‘No taxonomy’ (434 in our case) might be easier, as these encompass cases with inadequate taxonomy, i.e. typographic errors, interim species epithets or synonyms.

The number of new species records that could be added to BOLD based on BINs that were unidentified to a species level was 1,910 in July 2015 and had been reduced to 1,656 one year later (August 2016) (plus the 718 completely new-to-BOLD BINs). Thus 254 formerly unidentified BINs now have a species name, either through the BOLD reverse identification engine or expert data curation, efforts which we aim to increase through discussing these data in this descriptive article. To achieve this aim, our strategy involves sharing voucher specimens with the BFB- and GBOL-associated taxonomists to gain their advice (Geiger et al. 2016). Of special interest and first priority are singleton BINs, as they are likely to include a high proportion of very rare and cryptic species. We also invite the scientific community outside these initiatives to aid the identification of these specimens, which can be obtained from our collections (Suppl. material 2). Because most specimens were well-preserved as vouchers, it is also possible to check images of one or more individuals to gain a first taxonomic placement.

Since taxonomic coverage in the reference database may be biased towards organisms that are frequent or in high abundance, we expected a positive relation between level of taxonomic identification and prevalence and/or abundance. Judging from the top ten most abundant BINs in each trap (Tables 1, 2), this was not the case; five of the top ten BINs in the GMTPE sample were not resolved to a genus or species. In the GMTPZ sample the situation was different; three BINs showed an unambiguous species identification, but six BINs contained between 2 and 27 (!) different names in the global database. Cases such as BIN BOLD:ACD9582, where a species identification is lacking are certainly due to a shortage of taxonomists working on this group of Diptera. Other extreme cases, with up to 27 different names for a hoverfly (BOLD:AAA7374) suggest the need for a taxonomic revision of this almost globally detected lineage. The level of taxonomic identification and congruence for the 20 aforementioned BINs one year later (Oct 27th 2016) improved in 4 cases, remained the same in 14 BINs and decreased in 2 BINs, where even more taxa are listed within them (Tables 1, 2).

We emphasize that taxonomic decisions should be made within a comparative context, ideally including morphological data (cf. Jörger and Schrödl 2014) and also additional, independent genetic markers. If the decision to attach a specific taxon name to a specimen has been made solely based on reverse taxonomy, i.e. BIN membership interpreted as conspecificity, then this should be clearly indicated, as it is standard on BOLD: “Identifier: X. Y.”, “Identification Method: Tree Based Identification (March 2016)” or “Identification Method: BOLD ID Engine”.

## Conclusions

This study indicates the feasibility of developing and using new methods and standards for ecosystem management, and help to promote wide-scale screening of environmental samples for timely measurements of biodiversity. The urgent need to create such tools was recently considered at an official hearing of a committee of the German Parliament (Tumbrinck 2016). It reviewed data from a long-term monitoring program with Malaise traps at two locations in a nature conservation area in north-western Germany, which has revealed a 75% decrease in insect biomass between 1989 and 2013 (Sorg et al. 2013). The consequences of this massive decrease in just three decades cannot be properly evaluated as information on species composition is lacking for many taxonomic groups, because of the shortage of taxonomic specialists. Consequently, it is impossible to know which ecological functions have been lost or compromised. Our data show how this lack of knowledge can be overcome though traditional DNA barcoding approaches. High-throughput DNA sequencing provides another analytical option, which could make it possible to study the species compositions of massive arrays of Malaise traps. There is also a need to address the substantial gaps in the reference library, in particular for Diptera and Hymenoptera. As such progress will be impossible without the support of taxonomic experts, we invite members of this community to make use of the unidentified specimens (Suppl. material 2) encountered in this study in their own research.

Looking to the future, we hope that more countries will analyse their insect fauna using DNA barcoding. This will make it possible to draw significant conclusions by placing local results in a global context, insights which will become more tangible with broader participation. Other nations from Europe participating in the GMTP include Bulgaria, Finland, and Norway, but much denser sampling will be required to obtain a comprehensive picture of the regional or continental scale dynamics of biodiversity. An interesting and useful application will be to develop early warning systems, e.g. networks of continuous Malaise trap sampling on sensitive or critical access sites with automated notifications or flags when alien or invasive species are detected. Analogously, it will be helpful to create automated annotations for members of the IUCN or regional Red Lists as well as similar applications for pest species in forestry and agriculture. This could be relatively easy to achieve by generating regional species lists and also regional lists of known invasive species, which could be highlighted by implementing this service as a new feature on BOLD. When it comes to analysing environmental samples for species composition, we advocate the use of regional, well-curated custom DNA barcode reference libraries, ideally coupled with the involvement of taxonomists who can increase the identification success by examining those BINs, which show taxonomic discordance.

## Supplementary Material

Supplementary material 1 Result of the BIN discordance analysis (BOLD, September 25th 2015) for all BINs. Data type: Excel file with specimen details from the BIN discordance report
Brief description: Result of the BIN discordance analysis (BOLD, September 25th 2015).
File: oo_104105.xlsx Geiger, M., Rulik, B., Moriniere, J.

Supplementary material 2 Compilation of all specimens not identified to species level with embedded links leading to the respective BIN or specimen page on BOLD for an easy entry point to start refinements of taxonomic placements. Data type: Excel file
Brief description: Compilation of all specimens not identified to species level with embedded links leading to the respective BIN or specimen page on BOLD for an easy entry point to start refinements of taxonomic placements.
File: oo_104107.xlsx Geiger, M., Rulik, B., Moriniere, J.

## Figures and Tables

**Table 1.** Ten most common BINs in samples from GMTPE with BIN information from BOLD (Oct 21^st^ 2015 and Oct 27^th^ 2016): number of individuals and country of origin, taxonomy [number of species, if BIN discordant], occurrence in GMTPE samples and a general note. The hyperlink leads to the respective BIN page on BOLD with information on the geographical distribution of specimens and images of representatives.

**BIN**	**taxonomy Oct 21^st^ 2015**	**occurrence**	**Note (Oct 21^st^ 2015)**	**taxonomy Oct 27^st^ 2016**
BOLD:ACD9573 (n=1131, GER, NOR)	Diptera, Phoridae	May (395) Jun (156) Jul (281) Aug (145) Sep (129)	Species of phorid fly not yet morphologically determined	Metopininae, *Megaselia rufa* (n=1134)
BOLD:AAH3983 (n=4035, CAN, GER, NOR)	Diptera, Sciaridae, *Ctenosciara hyalipennis*	May (259) Jun (4) Jul (258) Aug (448) Sep (22)	*Ctenosciara* develops in decaying material	*Ctenosciara hyalipennis* *(n=4184; plus: SWE, FIN, UK)*
BOLD:ACF4704 (n=983, GER, BGR)	Diptera, Phoridae	May (21) Jun (110) Jul (453) Aug (292) Sep (92)	Species of phorid fly not yet morphologically determined	Metopininae, *Metopina oligoneura* (n=963)
BOLD:AAM9243 (n=1618, CAN, GER, NOR, FIN, NZL)	Diptera, Sciaridae, *Bradysia* spp. [2]	May (3) Jun (11) Aug (26) Sep (657)	Most likely *Bradysia polonica*	*Bradysia atroparva* (n=1659; plus RUS)
BOLD:ACE9016 (n=1165, GER, PAK, NOR, CHN, BGR, FIN, EGY, RUS, FRA, SAU, USA)	Diptera, Drosophilidae, Drosophilinae, *Scaptomyza pallida*	Jun (576) Jul (11) Aug (8) Sep (11)	Widely distributed in Mid-Europe, very common, larvae living saprohagic in rotting plant material	*Scaptomyza pallida* (n=1334;plus NLD, CZE, BGD, KEN, UK)
BOLD:ACD9582 (n=567, GER)	Diptera, Empididae	May (429) Jun (136) Jul (1)	Species of empidid fly not yet morphologically determined	Empididae (n=484)
BOLD:AAG7022 (n=553, GER, NOR, FIN, FRA)	Diptera, Phoridae, Metopininae, *Megaselia nigriceps*	Jun (108) Jul (190) Aug (65) Sep (177)	larvae living saprohagic in rotting plant material	*Megaselia nigriceps* (n=567)
BOLD:ACG4398 (n=373, GER, RUS)	Diptera, Chironomidae, Orthocladiinae, *Limnophyes* sp. 2SW	Jun (42) Jul (288) Aug (37) Sep (5)	A chironomid not yet morphologically determined	*Limnophyes* sp. 2SW (n=361)
BOLD:AAA8204 (n=1477, CAN, NOR, GER, FIN, BGR, FRA)	Diptera, Chironomidae, Orthocladiinae, *Limnophyes minimus*	May (65) Jun (86) Jul (144) Aug (47) Sep (27)	living almost everywhere in wet soils and at the margins of brooks and pools	*Limnophyes minimus* (n=1823; plus ARG, UK)
BOLD:ACG0645 (n=325, GER)	Diptera, Hybotidae	Jun (34) Jul (247) Aug (44) Sep (1)	Species of hybotid fly not yet morphologically determined	Tachydromiinae, *Drapetis* sp. (n=323)

**Table 2.** Ten most common BINs in samples from GMTPZ with BIN information from BOLD (Oct 21^st^ 2015 and Oct 27^th^ 2016): number of individuals and country of origin, taxonomy [number of species, if BIN discordant], occurrence in GMTPE samples and a general note. The hyperlink leads to the respective BIN page on BOLD with information on the geographical distribution of specimens and images of representatives.

**BIN**	**taxonomy Oct 21^st^ 2015**	**occurrence**	**note (Oct 21^st^ 2015)**	**taxonomy Oct 27^st^ 2016**
BOLD:ACR4672 (n=265, GER, BGR, EGY)	Diptera, Muscidae, Coenosiinae, *Coenosia testacea*	May (28) Jun (4) Jul (56) Aug (98) Sep (30)	Member of the greenhouse predator community (cf. http://www.studia-dipt.de/suppl9.htm)	*Coenosia testacea* (n=279; plus SWE)
BOLD:AAG9659 (n=1184, CAN, GER, NOR, BGR, FIN, FRA)	Diptera, Dolichopodidae, Diaphorinae, *Chrysotus* spp. [3]	Jun (29) Jul (118) Aug (47) Sep (9)	Member of the long-legged flies, known to be very abundant in e.g. wetlands (cf. Gelbič & Olejníček, 2011)	*Chrysotus neglectus* [1127] *Chrysotus femoratus* [2] *Chrysotus gramineus* [1] (n=1270; plus UK, USA, RUS)
BOLD:AAB4345 (n=251, GER, ITA, UK, FIN, ISR, NLD, AUT, ESP, DEN, NOR, CHN, GRL, BGR, GEO)	Lepidoptera, Noctuidae, Plusiinae, *Autographa* spp. [2]	Jul (68) Aug (105) Sep (13)	Most likely *A. gramma*, a polyphagous pest species found on cereals, grasses, fiber crops, *Brassica* spp., or other vegetables (cf. http://download.ceris.purdue.edu/file/2341)	*Autographa gramma* [80] *Autographa pulchrina* [1] *Lygephila craccae* [1] *Pingasa ruginaria* [1] (n=276; plus SWE, SRB, UAE, TUR, MDG, ROM, POR, KOR)
BOLD:ABV4597 (n=212, GER, FIN, BGR)	Diptera, Sarcophagidae, Sarcophaginae, *Sarcophaga* spp. [3]	May (2) Jun (45) Jul (106) Aug (8) Sep (24)	Most likely *S. depressifrons* in this megadiverse flesh-fly genus, where most species are scavengers of small carrion.	*Sarcophaga depressifrons* [207] *Sarcophaga haemorrhoa* [10] *Sarcophaga bulgarica* [1] (n=274; plus NOR, NLD)
BOLD:AAA7374 (n=1213, CAN, GER, BGR, FIN, NOR, PAK, EGY, USA, RUS, TUR, KEN, SUI, GRL, MAR, DEN, MYS, ESP, LBN, ITA, UK, SWE, NLD, CHN, SRB, KGZ)	Diptera, Syrphidae, Syrphinae, *Sphaerophoria* spp. [27]	May (2) Jun (11) Jul (92) Aug (40) Sep (9)	Most likely the very variable hoverfly *S. philanthus*, as frequent visitor of flowers a likely important pollinator species. The existence of 27 different names in this BIN certainly reflects the need of a revisionary work of this nearly globally occuring taxon.	*Sphaerophoria philanthus* [85] *Sphaerophoria scripta* [36] *Sphaerophoria batava* [28] *Sphaerophoria virgata* [20] *Sphaerophoria taeniata* [17] *Sphaerophoria interrupta* [16] *Sphaerophoria contigua* [12] *Sphaerophoria rueppellii* [9] *Sphaerophoria longipilosa* [8] *Sphaerophoria abbreviata* [8] *Sphaerophoria asymmetrica* [8] *Sphaerophoria laurae* [7] *Sphaerophoria sulphuripes* [7] *Sphaerophoria infuscata* [7] *Sphaerophoria bankowskae* [6] *Sphaerophoria fatarum* [5] *Sphaerophoria potentillae* [4] *Sphaerophoria bifurcata* [3] *Sphaerophoria brevipilosa* [3] *Sphaerophoria interrupta*-group [3] *Sphaerophoria pyrrhina* [3] *Sphaerophoria* sp. MA-1 [3] *Sphaerophoria boreoalpina* [2] *Sphaerophoria macrogaster* [2] *Sphaerophoria chongjini* [2] *Sphaerophoria* sp. [1] *Sphaerophoria bengalensis* [1] *Sphaerophoria kaa* [1] *Sphaerophoria cleoae* [1] Sphaerophoria nr. philanthus [1] (n=1590; plus FRA, JPN, TWN, AUS, IND, CZE, KOR, HUN, BGD)
BOLD:ACR3782 (n=223, GER, FIN)	Diptera, Muscidae, Coenosiinae, *Coenosia agromyzina*	May (33) Jun (20) Jul (21) Aug (31) Sep (33)	Member of the genus *Coenosia* have potential as biological control agents against plant pests due to their predatory way of life.	*Coenosia agromyzina* (n=246; plus SWE)
BOLD:AAB1062 (n=305, GER, FRA, RUS, UK, TUR, TJK, NOR, SWE, KGZ, SUI, UKR, ESP, ITA, CYP, IRN, CHL, IRL, MNG, AUT, LAT, GRE, DEN, NZL, ALG, POR, KOR, FIN, UZB, SLO, BEL)	Hymenoptera, Apidae, Apinae, *Bombus* spp. [15]	May (2) Jun (10) Jul (92) Aug (30) Sep (1)	Most likely *B. terrestris*, the very widespread European bumblebee with at least nine described subspecies. Despite numerous studies on this species complex there is no consens on the taxonomic status of several subspecies. (cf.	*Bombus terrestris* [208] *Bombus terrestris* subsp. [25] *Bombus maderensis* [2] *Bombus pascuorum* [1] *Bombus canariensis* [1] (n=361; plus NLD, EST)
BOLD:AAN8406 (n=254, GER, CAN, BGR, EGY, PAK)	Hemiptera, Cicadellidae, Typhlocybinae, *Empoasca* sp.	May (3) Jun (26) Jul (72) Aug (17) Sep (9)	A genus of leafhoppers with small sized species (ca. 3mm) of which some are considered destructive pests in field crops and vegetables in greenhouses.	*Empoasca pteridis* [3] *Empoasca vitis* [2] (n=392; plus NLD)
BOLD:AAC4378 (n=176, GER, NOR, ITA, FRA, IRL, BGR, FIN)	Hymenoptera, Apidae, Apinae, *Bombus* spp. [6]	May (11) Jun (27) Jul (17) Aug (37) Sep (8)	Most likely the common carder bee (*B. pascuorum*), a bumblebee present in most of Europe in various habitats.	*Bombus pascuorum* [73] Bombus pascuorum subsp. vulgo [2] *Bombus incertus* [1] *Bombus flavobarbatus* [1] (n=204; plus NLD, UK, DEN)
BOLD:ACR4546 (n=155, GER, NOR, FIN)	Diptera, Anthomyiidae, Pegomyinae, *Emmesomyia grisea*	May (9) Jun (17) Jul (49) Aug (6) Sep (8)	Most larvae of the species in the family dung flies are known as root maggots, but many larvae also feed on dung and animal feces. *Emmesomyia* are coprophagous and probably specific to cow droppings (cf. Iwasa 2007).	*Emmesomyia grisea* (n=185)
